# Identification of a disulfidptosis-related genes signature for diagnostic and immune infiltration characteristics in cervical cancer

**DOI:** 10.1371/journal.pone.0322387

**Published:** 2025-04-30

**Authors:** Qun Zhou, Bangli Song, Yibo He, Zhezhong Zhang, Shiliang Chen, Wenjun Chen, Xianbin Li, Jun Jiang

**Affiliations:** 1 Department of gynecology, The First Affiliated Hospital of Zhejiang Chinese Medical University (Zhejiang Provincial Hospital of Chinese Medicine), Hangzhou, Zhejiang, China; 2 Department of Internal Medicine, Zhejiang University of Technology Hospital, Hangzhou, Zhejiang, China; 3 Department of Clinical Lab, The First Affiliated Hospital of Zhejiang Chinese Medical University (Zhejiang Provincial Hospital of Chinese Medicine), Hangzhou, ZheJiang, China; 4 School of Nursing, Hangzhou Medical College, Hangzhou, Zhejiang, China; 5 School of Computer and Big Data Science, Jiujiang University, Jiujiang, China; The First Affiliated Hospital of Nanjing Medical University, CHINA

## Abstract

**Background:**

Cervical cancer (CC) ranks as the fourth most common malignancy affecting women globally, with research highlighting a rising incidence among younger age groups. Disulfidptosis, a newly identified form of regulated cell death, has been implicated in the pathogenesis of numerous diseases. This study employs bioinformatics analyses to explore the expression profiles and functional roles of disulfidptosis-related genes (DRGs) in the context of cervical cancer.

**Methods:**

Differential analysis of the gene expression matrix in CC was performed to identify differentially expressed genes. The overlap between these genes and disulfidptosis-related genes was then determined. Key hub genes were identified using multiple machine learning approaches, including LASSO regression, support vector machines (SVM), and random forest (RF). These hub genes were subsequently used to construct a predictive model, which was validated using external datasets to ensure robustness and reliability.

**Results:**

In this study, 11 overlapping genes were identified, among which four hub genes—BRK1, NDUFA11, RAC1, and NDUFS1—were extracted using machine learning techniques. The diagnostic performance of these hub genes was validated with external datasets, and a predictive model was constructed based on their expression. The model demonstrated an exceptionally high area under the curve (AUC) of 0.997. Moreover, AUC values exceeding 0.85 for two independent validation datasets further confirmed the model’s accuracy and stability. Notably, NDUFA11 and BRK1 showed significant associations with patient survival, highlighting their prognostic importance in cervical squamous cell carcinoma. Using CMAP and DGIdb databases, Metformin and Coenzyme-I were identified as potential targeted therapies for NDUFS1 and NDUFA11, respectively, offering new therapeutic avenues for patients.

**Conclusion:**

This study uncovered a strong association between disulfidptosis and CC and developed a predictive model to assess the risk in CC patients. These findings offer novel insights into identifying biomarkers and potential therapeutic targets for CC, paving the way for improved diagnostic and treatment strategies.

## Introduction

Cervical cancer (CC) is the fourth most commonly diagnosed malignancy among women worldwide. Alarmingly, recent studies indicate a rising incidence in younger populations, signaling a concerning trend [1,2]. With over 500,000 new cases reported annually, cervical cancer imposes a substantial global burden, profoundly affecting women’s health and creating significant challenges for economic development. Despite advancements in diagnostic techniques and cancer evaluation, identifying effective therapeutic targets and reliable molecular markers remains a critical and unmet need in the fight against this disease [3].

Disulfidptosis is a recently identified mode of cell death distinct from conventional forms such as apoptosis, necrosis, autophagy, netosis, and pyroptosis [4]. Liu et al. [5] demonstrated that cells overexpressing SLC7A11 undergo disulfidptosis under glucose-deprived conditions. This process is driven by the abnormal accumulation of disulfide molecules, which induces disulfide stress in actin cytoskeleton proteins. The resulting increase in disulfide bonds within actin filaments leads to filament contraction, structural disruption of the cytoskeleton, and ultimately, cell death. The excessive disulfide accumulation imposes mechanical stress on the actin cytoskeleton, causing its dysfunction and triggering this novel mechanism of cell death. Given the success of inhibitors targeting specific cell death pathways in treating diseases such as neurodegenerative disorders [6], the discovery of disulfidptosis presents a promising avenue for developing innovative therapeutic interventions [7].

Liu et al. [8] developed a predictive index based on long non-coding RNAs (lncRNAs) associated with disulfidptosis. This index holds significant clinical value by enhancing prognostic assessments for cervical cancer patients and serving as a biomarker to guide personalized treatment strategies. Jin et al. [9] confirmed the occurrence of disulfidptosis in cervical cancer cell lines by experimental validation. Guy et al. [10] indicated that SLC7A11 may serve as a valuable prognostic biomarker in cervical cancer, positively regulating the proliferation, migration, and invasion of cervical cancer cells. However, the relationship between disulfidptosis and CC remains largely unexplored, underscoring an urgent need to clarify its role in CC pathogenesis. Identifying disulfidptosis-related genes in CC could reveal novel biomarkers and therapeutic targets. For example, Wu et al. [11] employed bioinformatics methods to identify differentially expressed genes and pathways involved in CC progression, while Wang et al. [12] investigated genes and drugs associated with CC through bioinformatics analyses. Furthermore, Qi et al. [13] developed a miRNA-based prognostic signature for CC using an integrated bioinformatics framework. Despite these significant advancements, none of these studies have sufficiently examined the potential role of disulfidptosis in CC. Investigating this interplay is essential to bridging a critical knowledge gap and unlocking new therapeutic possibilities. Such research could not only deepen our understanding of CC pathogenesis but also pave the way for more effective treatment strategies.

This study aims to investigate the pathogenesis of CC through the lens of disulfidptosis. To identify and characterize differentially expressed disulfidptosis- related genes (de-DRGs) in CC, we conducted an initial screening using the GSE63514 dataset. These findings were further validated through analyses of the GSE67522 and GSE52903 datasets. By identifying potential biomarkers for CC therapy, our research seeks to enhance the understanding of CC pathogenesis and provide a foundation for the development of novel therapeutic strategies.

## Materials and methods

### Data acquisition and pre-processing

Three datasets of GSE63514, GSE67522, and GSE52903 were obtained from the GEO database using the “GEOquery” R package [14]. These datasets included gene expression profiles from both CC patients and healthy controls. Specifically, GSE63514 consisted of 28 CC samples and 24 normal samples; GSE67522 contained 18 CC samples and 11 control samples; and GSE52903 included 55 CC samples and 17 control samples. Sample pre-processing was performed in R, during which non-CC and abnormal control samples were excluded [15]. The detail of three datasets was in Table 1. Gene probes with null counts were removed, and duplicate probe expressions were averaged to ensure data quality. Additionally, a set of 27 genes strongly associated with disulfidptosis, as identified by Liu et al. [5] and Zheng et al [16], were included as key references. These genes—SLC7A11, SLC3A2, RPN1, NCKAP1, NUBPL, NDUFA11, LRPPRC, OXSM, NDUFS1, GYS1, MYH9, MYH10, MYL6, PRDX1, RAC1, DSTN, IQGAP1, CD2AP, ACTN4, PDLIM1, FLNB, ACTB, INF2, WASF2, CYFIP1, ABI2, and BRK1—served as a critical foundation for identifying de-DRGs in our study.

**Table 1:** The datasets of cervical cancer in this study.

Datasets	Tumor samples	Normal samples	Ref
GSE63514	28	24	[17]
GSE67522	18	11	[18]
GSE52903	55	17	[19]

### Identification of differentially expressed disulfidptosis-related genes (de-DEGs) associated with CC and disulfidptosis

The analysis of differential gene expression was conducted using the “limma” package. Fold change is a measure used to express the relative change between two conditions, typically in gene expression studies. It is calculated by dividing the value in one condition by the value in another. A fold change greater than 1 indicates an increase, while less than 1 indicates a decrease. For example, a fold change of 2 means a twofold increase, and 0.5 indicates a halving. Values close to 1 suggest minimal change and should be interpreted carefully, often requiring statistical analysis to confirm biological significance. The criterion for identifying DEGs between CC and normal samples was a | log2 fold change (FC)| > 0 and p < 0.05. We used volcano plots and heatmaps to display these results. To gain a deeper understanding of the biological functions of these DEGs, we utilized the “clusterProfiler” R package [20] to perform GO and KEGG enrichment analysis. Based on previous literature, 27 disulfidptosis-related genes (DRGs) were identified. By intersecting the DEGs with these DRGs, we pinpointed the differentially expressed disulfidptosis-related genes (de-DRGs).

### PPI network construction and correlation analysis of de-DRGs

The STRING database [21] was utilized to construct a schematic representation of the functional relationship network among the de-DRGs, referred to as the protein-protein interaction (PPI) network. Interactions with a medium confidence level, defined by a combined STRING score > 0.4, were included to ensure the inclusion of only experimentally validated and curated interactions. We used the STRING platform to visualize the PPI network. Additionally, the relationships between de-DRGs were further analyzed through Pearson correlation analysis, with the results visualized using the “corrplot” R package.

### Evaluating the immune cell infiltration

The “GSVA” R package [22] was used to perform single-sample gene set enrichment analysis (ssGSEA) to evaluate the degree of immune cell infiltration in each sample. The ssGSEA algorithm utilized 28 immune-related gene sets, encompassing genes associated with diverse immune cell types, functional activities, signaling pathways, and immune checkpoints. Using R packages (GSVA, GSEABase [23], and limma [24]), we applied the ssGSEA algorithm to systematically evaluate the immunological profiles of each sample included in the study. ssGSEA can quantify the activity of specific biological pathways or immune cell infiltration in individual samples, providing insights into tumor microenvironment characteristics and immune responses. For this purpose, 28 immune-related gene sets were established. The ssGSEA scores were derived from the expression matrix of each sample to quantify the level of immune cell infiltration. These analyses were carried out using the “IOBR” R package, which facilitated the assessment of immune cell infiltration levels across all samples.

### Construction of predictive model based on machine learning methods

A total of 27 DRGs were identified based on previous literature. By intersecting these DRGs with DEGs, we identified 11 de-DRGs. To refine the gene list for CC diagnosis, three methods based on machine learning were employed: LASSO, SVM, and RF. LASSO regression enhances prediction accuracy by selecting important feature variables through regularization techniques. SVM establishes a threshold between two classes to predict labels for feature vectors. RF excels at handling both classification and regression tasks, offering robust and reliable prediction results. Genes identified as significant across all three analyses (LASSO, SVM, and RF) were designated as central hub genes for CC diagnosis. Subsequently, we utilized a nomogram model in “rms” R package to predict CC cluster. The model’s ability to differentiate CC samples from normal samples was assessed through receiver operating characteristic (ROC) analysis with the “pROC” R package. Its diagnostic performance was further validated using ROC analysis on two independent external cervical tissue datasets.

### Verification of key genes via the Kaplan-Meier (KM) survival analysis based on TCGA database

The Kaplan-Meier (KM) method [25] was employed to assess the prognostic survival rates associated with key genes. For this analysis, the median expression values of the mRNAs were calculated. For this analysis, the median expression levels of the genes were calculated, with samples classified as high-expression if their gene levels exceeded the median, and low-expression if they were below it. To determine the relationship between these mRNAs and overall survival (OS), we utilized the TCGA-CESC database, which includes clinical information on patients. The hazard ratio (HR) and p-values were calculated to assess the direct correlation between gene expression and prognostic survival. A p-value of less than 0.05 was deemed statistically significant.

### Statistical analysis

Statistical analysis was performed using R software (version 3.6.2). The “limma” R package was utilized to identify DEGs between CC patients and normal samples, with a significance threshold set at p < 0.05.

## Results

### Identification of DEGs and functional and pathway enrichment analysis

**Fig 1:**
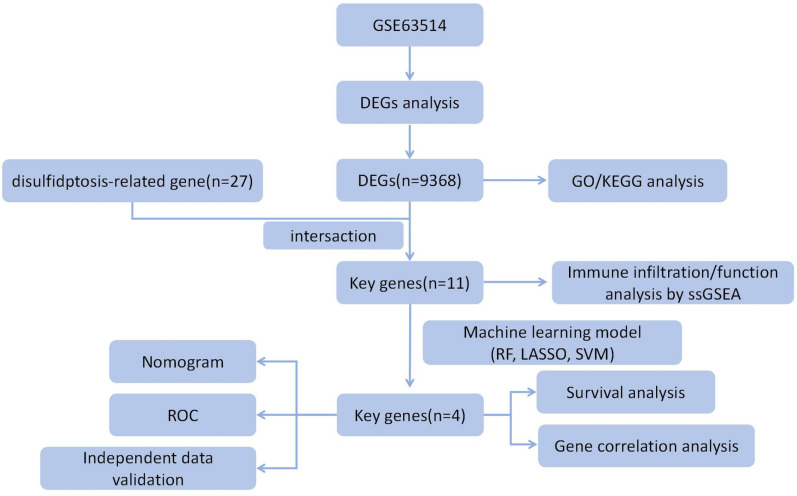
The study flow chart.

**Fig 2:**
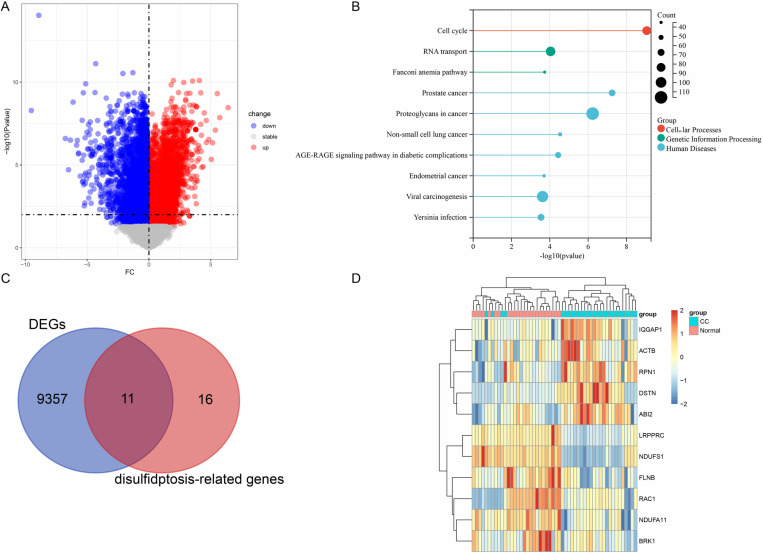
Differential expression analysis. (A) A volcano plot of the DEGs. (B) KEGG pathway enrichment analysis of DEGs. (C) A Venn diagram illustrating the overlap between DEGs and DRGs. (D) A heatmap depicting the expression of 11 de-DRGs based on the GSE63514 dataset.

The GSE63514 dataset included 28 CC patients and 24 normal samples. We identified a total of 9,368 DEGs by using the “limma” R package. The analysis workflow was detailed in Fig 1, with Fig 2A illustrating the volcano plot of the DEGs and Fig 2B showcasing the results of KEGG pathway enrichment analysis.

To investigate the potential function of these DEGs, we performed KEGG and GO enrichment analyses. The results indicated that these DEGs were significantly enriched in pathways, including the cell cycle, RNA transport, Fanconi anemia pathway, Yersinia infection, AGE-RAGE signaling pathway in diabetic complications, and viral carcinogenesis (Fig 2B). GO is divided into three categories: biological process (BP), cellular component (CC), and molecular function (MF). For BP, the significant terms were primarily associated with the positive regulation of biosynthetic processes, the positive regulation of nucleobase-containing metabolic processes, chromosome organization, cellular responses to DNA damage stimuli, and DNA replication. For CC, the DEGs were significantly enriched in nuclear protein complexes, nuclear bodies, centrosomes, nuclear envelopes, and chromosome centromeric regions. For MF, the significant terms were related to enzyme binding, ribonucleotide binding, cytoskeletal protein binding, chromatin binding, helicase activity, and DNA helicase activity.

These findings suggested that the identified DEGs play significant roles in CC and warrant further investigation. To identify de-DRGs, we conducted a cross-referencing analysis between the DEGs and a set of 27 genes previously associated with disulfidptosis. This intersection yielded 11 de-DRGs (Fig 2C). The expression patterns of these 11 de-DRGs in CC and normal samples were depicted in Fig 2D. Specifically, LRPPRC, NDUFS1, FLNB, RAC1, NDUFA11, and BRK1 showed lower expression levels in CC, whereas IQGAP1, ACTB, RPN1, DSTN, and ABI2 exhibited higher expression levels in CC. These results highlight the potential importance of these genes in CC pathogenesis and provide a foundation for further research.

### Construction of PPI network and correlation analysis of the 11 de-DRGs

**Fig 3:**
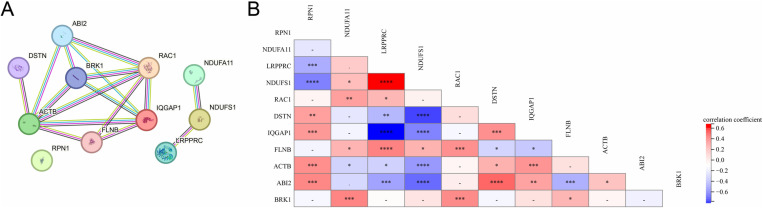
PPI network analysis (A) PPI network of 11 de-DRGs. (B) Pearson correlation analysis of the 11 de-DRGs.

To elucidate the relationships among these 11 de-DRGs, we performed PPI network analysis. The results revealed that these 11 de-DRGs are closely interconnected, forming a tightly linked network (Fig 3A). Notably, several hub genes-BRK1, RAC1, ABI2, ACTB, IQGAP1, DSTN, and FLNB were identified based on their high connectivity within the network. Additionally, to investigate potential correlations between the expression levels of these 11 de-DRGs, we performed correlation analysis. The results revealed that the majority of gene pairs displayed positive correlations, while only a few exhibited negative correlations (Fig 3B).

### Evaluation of immune cell infiltration

**Fig 4:**
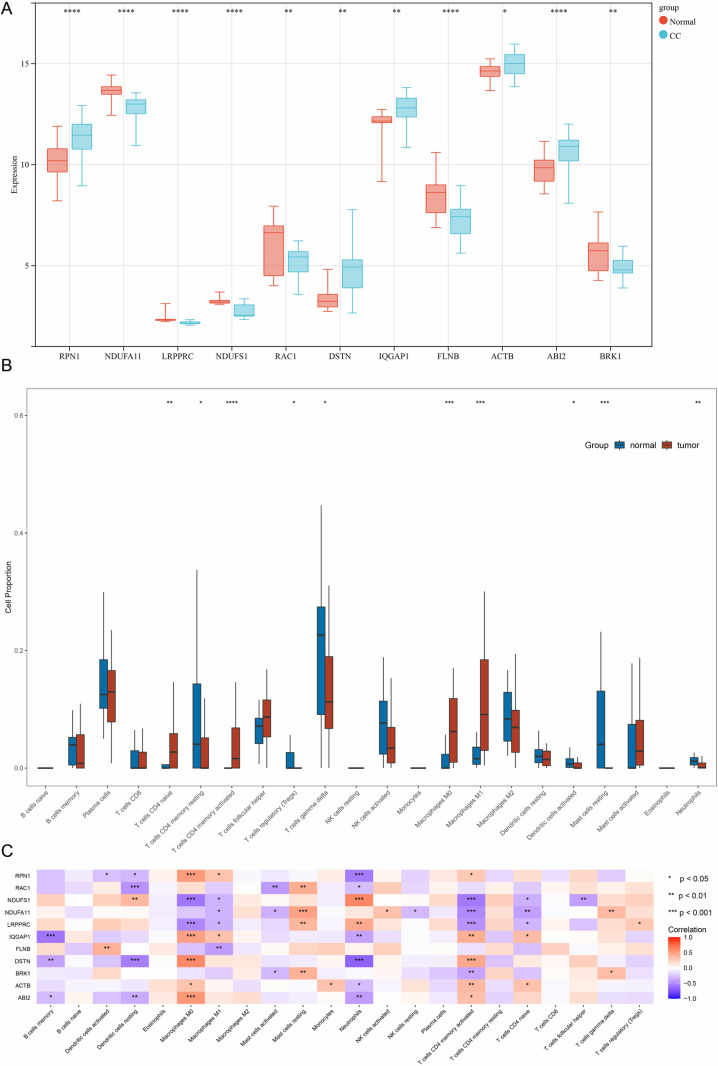
Immune infiltration of 11 de-DRGs. (A) Boxplot of 11 de-DRGs expression levels. (B) The fraction of immune cells comparison in CC and normal group. (C) The correlation between de-DRGs and immune cells.

We performed an immune infiltration analysis to examine potential differences in immune system activity between the CC and normal groups using the ssGSEA algorithm. Fig 4A illustrates the overall expression patterns of the 11 de-DRGs between CC and normal samples. The results identified significant variations in the proportions of 22 types of infiltrating immune cells between the two groups (Fig 4B). Specifically, the CC group exhibited elevated levels of activated CD4 + naive T cells, activated CD4 + memory T cells, M0 macrophages, and M1 macrophages. In contrast, the normal group demonstrated higher levels of resting CD4 + memory T cells, regulatory T cells, gamma delta T cells, activated dendritic cells, resting mast cells, and neutrophils. No significant differences were observed in the proportions of other immune cell types between the groups (Fig 4B).

To further elucidate the potential mechanisms by which de-DRGs impact the progression of CC, we investigated their associations with immune cells. Our analysis revealed that certain de-DRGs are closely linked to specific immune cell types. For instance, RPN1 exhibited a distinct positive correlation with M0 macrophages, M1 macrophages, and activated CD4 + memory T cells, while it showed negative correlations with activated dendritic cells, resting dendritic cells, and neutrophils. Similarly, IQGAP1, DSTN, ACTB, and ABI2 were positively correlated with M0 macrophages and activated CD4 + memory T cells but negatively correlated with memory B cells and neutrophils (Fig 4C). These findings indicate that distinct immune cell types exhibit unique infiltration patterns in CC patients, suggesting that de-DRGs play a pivotal role in shaping the immune microenvironment. This regulatory influence may have a significant impact on the progression of CC, underscoring the importance of these genes in advancing our understanding of the disease’s immunological mechanisms.

### Identification of the disulfidptosis-signature using machine learning

**Fig 5:**
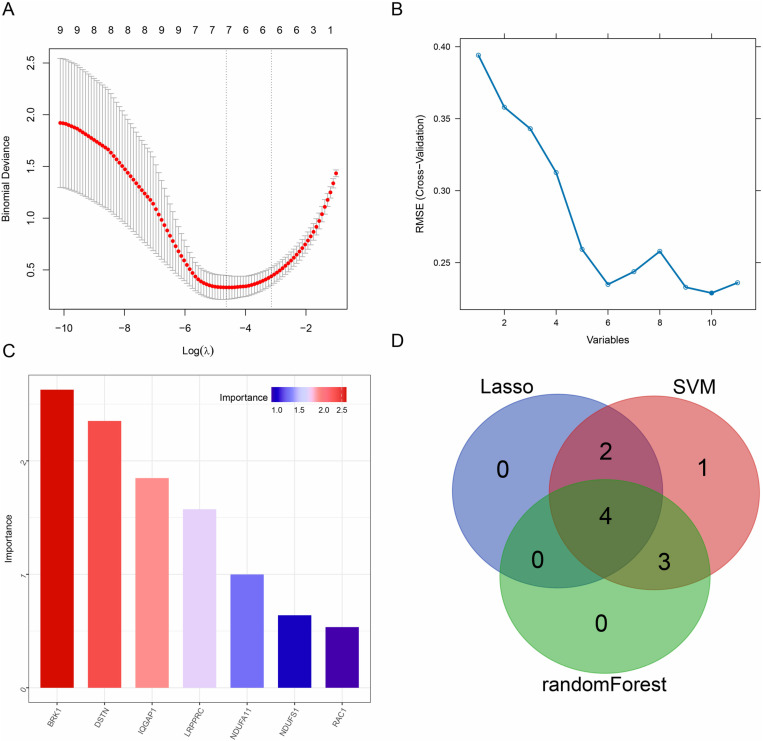
Screen of disulfidptosis-related signatures. (A) LASSO regression. (B) SVM. (C) RF. (D) A Venn diagram illustrates the intersection of candidate genes identified by the three methods.

Starting with the 11 identified de-DRGs, we utilized LASSO regression, SVM, and RF algorithms to screen potential candidates and establish a disulfidptosis-related gene signature (Figs 5A-C). The results revealed that 6, 7, and 8 genes were identified using the LASSO, SVM, and RF methods, respectively. This integrative multi-algorithm strategy ultimately pinpointed four hub genes associated with disulfidptosis: BRK1, NDUFA11, RAC1, and NDUFS1 (Fig 5D).

**Fig 6:**
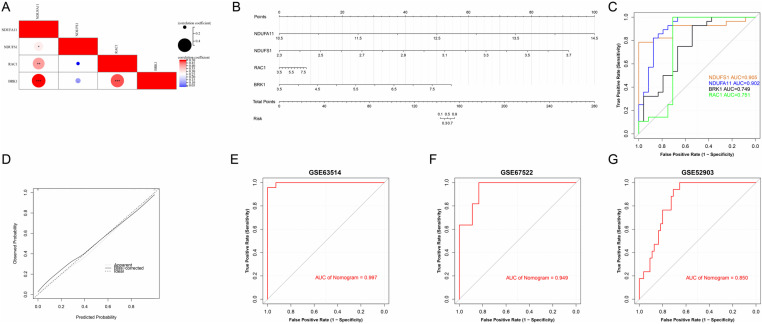
Validation of the diagnostic efficacy of hub genes. (A) Correlation analysis of the four hub genes reveals their relationships, with positive correlations shown in red and negative correlations in blue. (B) A nomogram was constructed to predict the risk of CC based on the identified hub genes. (C) ROC curves were generated to evaluate the diagnostic performance of the hub genes in CC. (D) A calibration curve was used to assess the prediction accuracy of the nomogram model. (E-G) ROC curves from external validation datasets, including GSE63514, GSE67522, and GSE52903, respectively, further confirmed the diagnostic efficacy of the model.

Most of the hub genes exhibited strong correlations with each other, particularly BRK1, which showed a robust synergistic relationship with NDUFA11. In contrast, RAC1 demonstrated a strong antagonistic relationship with NDUFS1 (Fig 6A). To assess the diagnostic potential of individual feature genes in predicting CC, we conducted a receiver operating characteristic (ROC) curve analysis and developed a nomogram model as a predictive tool (Fig 6B). The area under the curve (AUC) values for the ROC curves of NDUFS1, NDUFA11, BRK1, and RAC1 were 0.905, 0.902, 0.749, and 0.751, respectively (Fig 6C), indicating strong discriminatory power for the first two genes. The calibration curve demonstrated minimal discrepancy between predicted and actual risks, highlighting the high predictive accuracy of the nomogram model (Fig 6D). Notably, the nomogram achieved an AUC of 0.997, reflecting near-perfect diagnostic performance (Fig 6E).

External validation using independent datasets GSE67522 and GSE52903 further corroborated the diagnostic value of these hub genes, with AUCs of 0.949 and 0.850, respectively (Figs 6F and 6G). Collectively, these findings confirm that the nomogram model offers exceptional diagnostic accuracy, effectively distinguishing CC patients from healthy controls.

### Verification of hub genes via the Kaplan-Meier (KM) survival analysis

**Fig 7:**
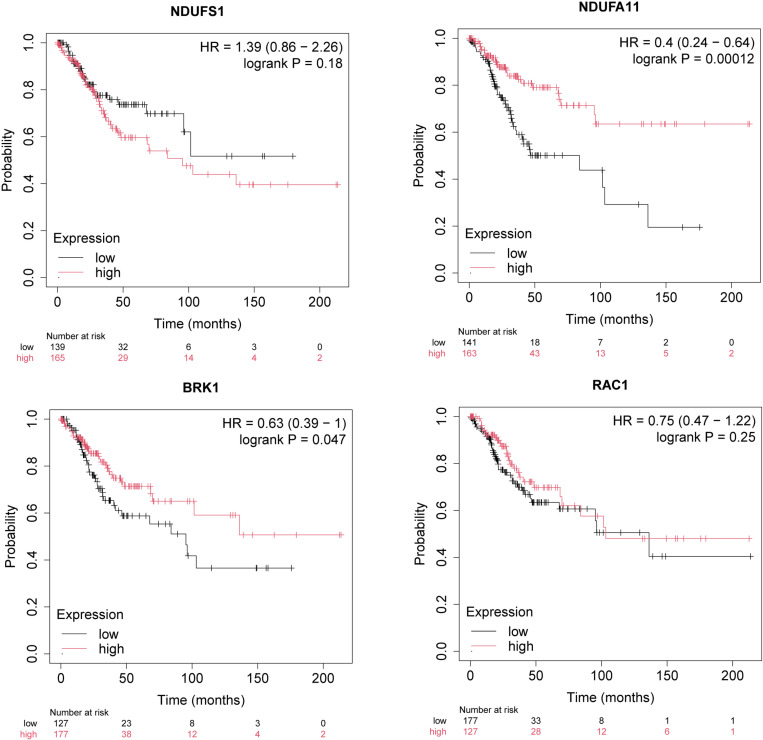
The Kaplan-Meier survival analysis of four genes extracted from CESC.

Among the four identified hub genes, NDUFA11 and BRK1 exhibited significant variations in patient survival, impacting the prognostic outcomes of patients with cervical squamous cell carcinoma (CESC) (Fig 7). These genes can be categorized into two groups based on their effects on survival: 1) with positive impacts: NDUFA11 (HR = 0.4, log_rank p = 0.00012); BRK1 (HR = 0.63, log_rank p = 0.047); RAC1 (HR = 0.75, log_rank p = 0.25). 2) with negative impacts: NDUFS1 (HR = 1.39, log_rank p = 0.18). Although RAC1 and NDUFS1 did not reach statistical significance in the survival analysis, our findings suggest that all four genes are closely associated with cervical cancer (CC). To our knowledge, this is one of the first studies to uncover these associations, highlighting the need for further experimental validation to explore their roles in CC prognosis.

### Prediction of potential therapeutic drugs

To identify potential candidate agents for NDUFS1, NDUFA11, BRK1, and RAC1, we utilized the Drug-Gene Interaction Database (DGIbd) [26] and the Connectivity Map (CMAP) database [27]. According to these databases: For NDUFS1 and NDUFA11, Metformin and Coenzyme-I were identified as potential targeted therapies, respectively. For RAC1, Azathioprine, Vemurafenib, Dabrafenib, and Dextromethorphan were identified as potential targeted medicines. These findings are summarized in Table 2.

**Table 2:** Prediction of potential therapeutic drugs.

Drug	Gene	Source	PubChem	Ref
Metformin	NDUFS1	DGIbd	14219	[28]
Coenzyme-l	NDUFS1	CMAP	439153	[34]
Metformin	NDUFA11	DGIbd	14219	[29]
Coenzyme-l	NDUFA11	CMAP	439153	[30]
Azathioprine	RAC1	DGIbd	2265	[35]
Vemurafenib	RAC1	DGIbd	42611257	–
Dabrafenib	RAC1	DGIbd	44462760	–
Dextromethorphan	RAC1	CMAP	5360696	–

Chen et al. [28] reported that metformin enhances the anticancer efficacy of everolimus in CC, proposing a combination therapy with these drugs as a promising new treatment strategy for patients. In a related study, Chen et al. [29] demonstrated that metformin markedly reduces cell viability and migration while promoting apoptosis and inducing cell cycle arrest in human cervical cancer cell lines. Additionally, Li et al. [30] identified Acetyl-CoA Synthase 2 as a potential biomarker for the diagnosis and prognosis of cervical squamous cell carcinoma (CESC). Wang et al. [31] found that combining dextromethorphan with metformin shows significant preventive and therapeutic potential against nicotine-induced esophageal squamous cell carcinoma (ESCC). While this research pertains specifically to ESCC, it hints at the broader applicability of such drug combinations in oncology. Mazieres et al. [32] noted that Vemurafenib monotherapy is efficacious for patients with BRAFV600-mutated non-small cell lung cancer (NSCLC), but not for those with BRAFnonV600 mutations. Kelly et al. [33] established that the duo of Dabrafenib and Trametinib offers an effective treatment strategy for NSCLC. To date, there is limited evidence directly linking Vemurafenib, Dabrafenib, and Dextromethorphan to cervical cancer treatment. However, based on the predictions from our study, these drugs may hold untapped potential for managing cervical cancer, underscoring the need for further investigation into their efficacy in this context.

## Discussion

Cervical cancer (CC) remains a significant health challenge for women globally, with its high mortality rate largely attributable to delayed diagnosis. Early detection through specific biomarkers is crucial for improving patient prognosis. Several studies have demonstrated that genes exhibiting partial disorder in CC patients can serve as valuable diagnostic biomarkers [36,37]. However, the current therapeutic landscape for CC remains suboptimal due to insufficient markers and the disease’s heterogeneous pathogenesis [38,39]. Consequently, identifying more effective diagnostic markers is essential for guiding personalized treatment strategies. Disulfidptosis, a recently discovered form of cell death driven by the accumulation of disulfide bonds, leads to cytoskeletal collapse and subsequent cell death, playing a potential role in disease progression [40]. However, the regulatory mechanisms and involvement of disulfidptosis in various diseases remain insufficiently understood, and the pathways underlying this process require further exploration. This study focuses on elucidating the role of disulfidptosis-related genes in CC. By employing comprehensive bioinformatics analyses, we aim to establish a connection between disulfidptosis and the pathogenesis of CC, identifying key genes associated with this mechanism. Our findings aim to provide novel insights and identify potential therapeutic targets to enhance understanding and treatment of cervical cancer.

In this study, we analyzed gene expression profiles from the GEO database to compare normal and CC patients, identifying a total of 9,368 DEGs. GO and KEGG enrichment analyses revealed significant enrichment in several biological processes and pathways, including the cell cycle, RNA transport, Fanconi anemia pathway, Yersinia infection, AGE-RAGE signaling pathway in diabetic complications, and viral carcinogenesis. These findings align with previous studies highlighting cell cycle dysfunction as a hallmark of CC [41–43]. Notably, the Fanconi anemia pathway and RNA transport were identified as potential therapeutic targets for CC, offering valuable insights for future research and treatment strategies [44,45].

Subsequently, we conducted a comprehensive evaluation of the expression of de-DRGs in both normal and CC individuals. The results revealed significantly abnormal de-DRG expression in CC patients compared to the normal population, highlighting the critical role of DRGs in CC progression. Furthermore, we observed substantial changes in immune cell composition between normal and CC patients. Consistent with previous studies demonstrating the strong association between the immune system and CC [46,47], our findings revealed higher infiltration of activated T cells CD4 naïve, T cells CD4 memory activated, macrophages M0, and macrophages M1 in CC patients, aligning with prior research and reinforcing the importance of immune modulation in CC pathogenesis [48–50].

Additionally, we identified a correlation between immune cells and disulfidptosis-related genes (DRGs). Using three machine learning classifiers, we further identified four hub genes—BRK1, NDUFA11, RAC1, and NDUFS1—that are associated with CC. The diagnostic performance of these hub genes was validated through external datasets and clinical samples. BRK1, located at 3p25.3, is a subunit of the SCAR/WAVE actin nucleating complex involved in Rac protein signal transduction and the positive regulation of cellular component organization. Li et al. [51] found that BRK1 expression levels were significantly associated with CC, particularly in specific immune cell types. NDUFA11, a protein-coding gene for NADH: Ubiquinone Oxidoreductase Subunit A11, is linked to mitochondrial complex I deficiencies, including Mitochondrial Complex I Deficiency, Nuclear Type 14, and Isolated Complex I Deficiency. Related pathways include respiratory electron transport, ATP synthesis via chemiosmotic coupling, heat production by uncoupling proteins, and complex I biogenesis. Kumar et al. [52] demonstrated that NDUFA11 (metabolic enzymes) was associated with cervical cancer. RAC1, which contains a nuclear localization signal (NLS) [53], has been shown to undergo nuclear import mediated by the importin Karyopherin alpha 2 (KPNA2), which interacts with the NLS. This process requires RAC1 activation. Mendoza et al. [54] demonstrated that RAC1 is expressed in the nucleus of epithelial cells in squamous intraepithelial lesions (SILs) and cervical cancer cell lines, and that chemical inhibition of RAC1 reduces cellular proliferation. NDUFS1, a protein-coding gene for NADH: Ubiquinone Oxidoreductase Core Subunit S1, is associated with mitochondrial complex V deficiency, complex I deficiency. Although studies linking NDUFS1 directly to CC are lacking, research has shown its role in promoting colorectal cancer cell proliferation and tumorigenesis. Our correlation analysis revealed significant synergistic and antagonistic interactions among these four hub genes. Building a diagnostic model using these genes may provide valuable insights for the clinical diagnosis of CC, offering a promising approach for improving diagnostic accuracy in clinical practice.

This study has several limitations that warrant emphasis. Firstly, our research primarily relies on comprehensive bioinformatics analysis and other datasets validation, lacking direct experimental and clinical trial data to confirm our findings. Therefore, further studies are necessary to validate our results through rigorous laboratory experiments and clinical trials. Additionally, the sample size in this study is relatively modest, which may limit the generalizability of our findings. Larger-scale studies are essential to verify the reliability and robustness of our results. Moreover, this investigation focused on a single cohort of cervical cancer (CC) patients and controls, without accounting for potential confounding factors such as age, sex, and ethnicity. Future studies should incorporate diverse populations to better understand how these variables might influence the outcomes. Finally, expanding the number of CC samples will be vital for refining the clustering related to disulfidptosis and confirming its accuracy. Enhanced sampling will provide a more comprehensive view of the disease’s heterogeneity and improve the precision of our analytical models. In summary, while our study provides valuable insights into the role of disulfidptosis-related genes in cervical cancer, addressing these limitations through future research will strengthen the validity and applicability of our findings.

## Supporting information

S1 File The dataset (GSE63514) used in this study. (GZ)

## References

[1] Siegel RL, Miller KD, Jemal A. Cancer statistics, 2020. CA Cancer J Clin. 2020;70(1):7–30. doi: 10.3322/caac.21590 31912902

[2] Gupta S, Harper A, Ruan Y, Barr R, Frazier AL, Ferlay J, et al. International Trends in the Incidence of Cancer Among Adolescents and Young Adults. J Natl Cancer Inst. 2020;112(11):1105–17. doi: 10.1093/jnci/djaa007 32016323 PMC7669231

[3] Rahangdale L, Mungo C, O’Connor S, Chibwesha CJ, Brewer NT. Human papillomavirus vaccination and cervical cancer risk. BMJ. 2022;379:e070115. Epub 20221215. doi: 10.1136/bmj-2022-070115 36521855

[4] Jorgensen I, Zhang Y, Krantz BA, Miao EA. Pyroptosis triggers pore-induced intracellular traps (PITs) that capture bacteria and lead to their clearance by efferocytosis. J Exp Med. 2016;213(10):2113–28. Epub 20160829. doi: 10.1084/jem.20151613 27573815 PMC5030797

[5] Liu X, Nie L, Zhang Y, Yan Y, Wang C, Colic M, et al. Actin cytoskeleton vulnerability to disulfide stress mediates disulfidptosis. Nat Cell Biol. 2023;25(3):404–14. Epub 20230206. doi: 10.1038/s41556-023-01091-2 36747082 PMC10027392

[6] Deng L, He S, Guo N, Tian W, Zhang W, Luo L. Molecular mechanisms of ferroptosis and relevance to inflammation. Inflamm Res. 2023;72(2):281–99. doi: 10.1007/s00011-022-01672-1 36536250 PMC9762665

[7] Machesky LM. Deadly actin collapse by disulfidptosis. Nat Cell Biol. 2023;25(3):375–6. doi: 10.1038/s41556-023-01100-4 36918690

[8] Liu L, Liu J, Lyu Q, Huang J, Chen Y, Feng C, et al. Disulfidptosis-associated LncRNAs index predicts prognosis and chemotherapy drugs sensitivity in cervical cancer. Sci Rep. 2023;13(1):12470. Epub 20230801. https://doi.org/10.1038/s41598-023-39669-3 37528124 10.1038/s41598-023-39669-3 PMC10394072

[9] Jin T, Yin T, Xu R, Liu H, Yuan S, Xue Y, et al. Exploring the role of disulfidptosis-related signatures in immune microenvironment, prognosis and therapeutic strategies of cervical cancer. Transl Oncol. 2024;44:101938. doi: 10.1016/j.tranon.2024.101938 38492499 PMC10955422

[10] Guy MM, Bian T, Sun L, Hao Y, Jiao X, Zhang W, et al. SLC7A11 is a potential therapeutic target and prognostic biomarker correlated with immune cell infiltration in cervical cancer. Discov Oncol. 2025;16(1):125. Epub 20250206. doi: 10.1007/s12672-025-01888-7 39915437 PMC11802985

[11] Wu B, Xi S. Bioinformatics analysis of differentially expressed genes and pathways in the development of cervical cancer. BMC Cancer. 2021;21(1):733. 20210626. doi: 10.1186/s12885-021-08412-4 34174849 PMC8236200

[12] Wang D, Liu Y, Cheng S, Liu G. Identification of Novel Genes and Associated Drugs in Cervical Cancer by Bioinformatics Methods. Med Sci Monit. 2022;28:e934799. doi: 10.12659/msm.934799 35428744 PMC9020271

[13] Qi Y, Lai Y-L, Shen P-C, Chen F-H, Lin L-J, Wu H-H, et al. Identification and validation of a miRNA-based prognostic signature for cervical cancer through an integrated bioinformatics approach. Sci Rep. 2020;10(1):22270. Epub 20201217. doi: 10.1038/s41598-020-79337-4 33335254 PMC7747620

[14] Davis S, Meltzer PS. GEOquery: a bridge between the Gene Expression Omnibus (GEO) and BioConductor. Bioinformatics. 2007;23(14):1846–7. doi: 10.1093/bioinformatics/btm254 17496320

[15] Liang G, Cao W, Tang D, Zhang H, Yu Y, Ding J, et al. Nanomedomics. ACS Nano. 2024;18(17):10979–1024. Epub 20240418. doi: 10.1021/acsnano.3c11154 38635910

[16] Zheng P, Zhou C, Ding Y, Duan S. Disulfidptosis: a new target for metabolic cancer therapy. J Exp Clin Cancer Res. 2023;42:103. doi: 10.1186/s13046-023-02675-4 37101248 PMC10134647

[17] den Boon JA, Pyeon D, Wang SS, Horswill M, Schiffman M, Sherman M, et al. Molecular transitions from papillomavirus infection to cervical precancer and cancer: Role of stromal estrogen receptor signaling. Proc Natl Acad Sci U S A. 2015;112(25):E3255-64. doi: 10.1073/pnas.1509322112 26056290 PMC4485108

[18] Sharma S, Mandal P, Sadhukhan T, Roy Chowdhury R, Ranjan Mondal N, Chakravarty B, et al. Bridging Links between Long Noncoding RNA HOTAIR and HPV Oncoprotein E7 in Cervical Cancer Pathogenesis. Sci Rep. 2015;5:11724. doi: 10.1038/srep11724 26152361 PMC4495428

[19] Medina-Martinez I, Barrón V, Roman-Bassaure E, Juárez-Torres E, Guardado-Estrada M, Espinosa AM, et al. Impact of gene dosage on gene expression, biological processes and survival in cervical cancer: a genome-wide follow-up study. PLoS One. 2014;9(5):e97842. doi: 10.1371/journal.pone.0097842 24879114 PMC4039463

[20] Yu G, Wang L-G, Han Y, He Q-Y. clusterProfiler: an R package for comparing biological themes among gene clusters. OMICS. 2012;16(5):284–7. doi: 10.1089/omi.2011.0118 22455463 PMC3339379

[21] Szklarczyk D, Gable AL, Lyon D, Junge A, Wyder S, Huerta-Cepas J, et al. STRING v11: protein-protein association networks with increased coverage, supporting functional discovery in genome-wide experimental datasets. Nucleic Acids Res. 2019;47(D1):D607–13. Epub 2018/11/27. doi: 10.1093/nar/gky1131 30476243 PMC6323986

[22] Hänzelmann S, Castelo R, Guinney J. GSVA: gene set variation analysis for microarray and RNA-seq data. BMC Bioinformatics. 2013;14:7. doi: 10.1186/1471-2105-14-7 23323831 PMC3618321

[23] Jin Y, Wang Z, He D, Zhu Y, Chen X, Cao K. Identification of novel subtypes based on ssGSEA in immune-related prognostic signature for tongue squamous cell carcinoma. Cancer Med. 2021;10(23):8693–707. doi: 10.1002/cam4.4341 34668665 PMC8633230

[24] Ritchie ME, Phipson B, Wu D, Hu Y, Law CW, Shi W, et al. limma powers differential expression analyses for RNA-sequencing and microarray studies. Nucleic Acids Res. 2015;43(7):e47. doi: 10.1093/nar/gkv007 25605792 PMC4402510

[25] Saluja R, Cheng S, delos Santos KA, Chan KKW. Estimating hazard ratios from published Kaplan‐Meier survival curves: A methods validation study. Research Synthesis Methods. 2019;10(3):465–75. doi: 10.1002/jrsm.1362 31134735

[26] Wagner AH, Coffman AC, Ainscough BJ, Spies NC, Skidmore ZL, Campbell KM, et al. DGIdb 2.0: mining clinically relevant drug-gene interactions. Nucleic Acids Res. 2016;44(D1):D1036-44. doi: 10.1093/nar/gkv1165 26531824 PMC4702839

[27] Lamb J, Crawford ED, Peck D, Modell JW, Blat IC, Wrobel MJ, et al. The Connectivity Map: using gene-expression signatures to connect small molecules, genes, and disease. Science. 2006;313(5795):1929–35. doi: 10.1126/science.1132939 17008526

[28] Chen Y-H, Wu J-X, Yang S-F, Chen M-L, Chen T-H, Hsiao Y-H. Metformin Potentiates the Anticancer Effect of Everolimus on Cervical Cancer In Vitro and In Vivo. Cancers (Basel). 2021;13(18). Epub 2021 Sep 14. doi: 10.3390/cancers13184612 34572837 PMC8468269

[29] Chen Y-H, Yang S-F, Yang C-K, Tsai H-D, Chen T-H, Chou M-C, et al. Metformin induces apoptosis and inhibits migration by activating the AMPK/p53 axis and suppressing PI3K/AKT signaling in human cervical cancer cells. Mol Med Rep. 2021;23(1):88. Epub 20201125. doi: 10.3892/mmr.2020.11725 33236135 PMC7716426

[30] Li C-J, Chiu Y-H, Chang C, Chang Y-I, Sheu JJ, Chiang A-J. Acetyl Coenzyme A Synthase 2 Acts as a Prognostic Biomarker Associated with Immune Infiltration in Cervical Squamous Cell Carcinoma. Cancers (Basel). 2021;13(13):3125. doi: 10.3390/cancers13133125 34206705 PMC8269092

[31] Wang L, Du L, Xiong X, Lin Y, Zhu J, Yao Z, et al. Repurposing dextromethorphan and metformin for treating nicotine-induced cancer by directly targeting CHRNA7 to inhibit JAK2/STAT3/SOX2 signaling. Oncogene. 2021;40(11):1974–87. doi: 10.1038/s41388-021-01682-z 33603170 PMC7979537

[32] Mazieres J, Cropet C, Montané L, Barlesi F, Souquet PJ, Quantin X, et al. Vemurafenib in non-small-cell lung cancer patients with BRAFV600 and BRAFnonV600 mutations. Ann Oncol. 2020;31(2):289–94. doi: 10.1016/j.annonc.2019.10.022 31959346

[33] Kelly RJ. Dabrafenib and trametinib for the treatment of non-small cell lung cancer. Expert Rev Anticancer Ther. 2018;18(11):1063–8. doi: 10.1080/14737140.2018.1521272 Epub 30198802

[34] Palan PR, Mikhail MS, Shaban DW, Romney SL. Plasma concentrations of coenzyme Q10 and tocopherols in cervical intraepithelial neoplasia and cervical cancer. Eur J Cancer Prev. 2003;12(4):321–6. doi: 10.1097/00008469-200308000-00013 12883386

[35] Balachandran I, Galagan KS. Cervical carcinoma in situ associated with azathioprine therapy. A case report and literature review. Acta Cytol. 1984;28(6):699–702. 6391055

[36] Han L, Husaiyin S, Ma C, Wang L, Niyazi M. TNFAIP8L1 and FLT1 polymorphisms alter the susceptibility to cervical cancer amongst uyghur females in China. Biosci Rep. 2019;39(7):BSR20191155. doi: 10.1042/bsr20191155 31289124 PMC6639457

[37] Apu MNH, Aktar MN, Rahman MM, Mostaid MS. Association of TGFB1 gene polymorphisms with cervical cancer in Bangladeshi women: A case-control study. Tumour Biol. 2021;43(1):27–35. doi: 10.3233/tub-200061 33935123

[38] Sharma S, Deep A, Sharma AK. Current Treatment for Cervical Cancer: An Update. Anticancer Agents Med Chem. 2020;20(15):1768–79. doi: 10.2174/1871520620666200224093301 32091347

[39] Jin J, Fan Z, Long Y, Li Y, He Q, Yang Y, et al. Matrine induces ferroptosis in cervical cancer through activation of piezo1 channel. Phytomedicine. 2024;122:155165. doi: 10.1016/j.phymed.2023.155165 37922791

[40] Chen H, Yang W, Li Y, Ma L, Ji Z. Leveraging a disulfidptosis-based signature to improve the survival and drug sensitivity of bladder cancer patients. Front Immunol. 2023;14:1198878. doi: 10.3389/fimmu.2023.1198878 Epub 20230530 37325625 PMC10266281

[41] Wu X, Peng L, Zhang Y, Chen S, Lei Q, Li G, et al. Identification of Key Genes and Pathways in Cervical Cancer by Bioinformatics Analysis. Int J Med Sci. 2019;16(6):800–12. Epub 20190602. https://doi.or/10.7150/ijms.34172 31337953 10.7150/ijms.34172 PMC6643108

[42] Guo Y, Luo S. miR‑140‑5p inhibits cervical cancer cell phenotypes via downregulating FEN1 to halt the cell cycle. Mol Med Rep. 2020;22(6):4919–30. doi: 10.3892/mmr.2020.11552 Epub 20200930 33173950

[43] He M, Wang Y, Cai J, Xie Y, Tao C, Jiang Y, et al. LncRNA DLEU2 promotes cervical cancer cell proliferation by regulating cell cycle and NOTCH pathway. Exp Cell Res. 2021;402(1):112551. Epub 20210304. doi: 10.1016/j.yexcr.2021.112551 33675808

[44] Wang S, Ding B, Cui M, Yan W, Xia Q, Meng D, et al. Fanconi Anemia Pathway Genes Advance Cervical Cancer via Immune Regulation and Cell Adhesion. Front Cell Dev Biol. 2021;9:734794. Epub 20211115. doi: 10.3389/fcell.2021.734794 34869316 PMC8634638

[45] He W, Hong X, Chen G, Luo X, Lin Y. RNA modifications in gynecological cancer: current status and future directions. Front Med (Lausanne). 2024;11:1314075. doi: 10.3389/fmed.2024.1314075 38343637 PMC10853395

[46] Linares I, Berenguer Frances M, Cañas R, Najjari D, Gutiérrez C, Marín S, et al. Brachytherapy for targeting the immune system in cervical cancer patients. BMC Immunol. 2023;24(1):23. Epub 20230809. doi: 10.1186/s12865-023-00559-y 37559025 PMC10413692

[47] Avisha M, Pelupessy NU, Rahman A, Rauf S, Rakhmah N, Hamid F. Pre-treatment inflammatory and immune system parameters predicting cervical cancer metastasis. Turk J Obstet Gynecol. 2023;20(4):285–92. doi: 10.4274/tjod.galenos.2023.80912 38073222 PMC10711526

[48] Chen H, Ma R, Zhou B, Yang X, Duan F, Wang G. Integrated immunological analysis of single-cell and bulky tissue transcriptomes reveals the role of interactions between M0 macrophages and naïve CD4+ T cells in the immunosuppressive microenvironment of cervical cancer. Comput Biol Med. 2023;163:107151. doi: 10.1016/j.compbiomed.2023.107151 37348264

[49] Wang F, Yue S, Huang Q, Lei T, Li X, Wang C, et al. Cellular heterogeneity and key subsets of tissue-resident memory T cells in cervical cancer. NPJ Precis Oncol. 2024;8(1):145. Epub 20240716. doi: 10.1038/s41698-024-00637-3 39014148 PMC11252146

[50] Wang Q, Sudan K, Schmoeckel E, Kost BP, Kuhn C, Vattai A, et al. CCL22-Polarized TAMs to M2a Macrophages in Cervical Cancer In Vitro Model. Cells. 2022;11(13):2027. doi: 10.3390/cells11132027 35805111 PMC9265611

[51] Li N, Yi H, Sun W, Sundquist J, Sundquist K, Zhang X, et al. Revealing genes associated with cervical cancer in distinct immune cells: A comprehensive Mendelian randomization analysis. Int J Cancer. 2024;155(1):149–58. Epub 20240306. doi: 10.1002/ijc.34911 38447016

[52] Kumar K, Bose S, Chakrabarti S. Identification of Cross-Pathway Connections via Protein-Protein Interactions Linked to Altered States of Metabolic Enzymes in Cervical Cancer. Front Med (Lausanne). 2021;8:736495. doi: 10.3389/fmed.2021.736495 Epub 20211101 34790674 PMC8591138

[53] Lanning CC, Ruiz-Velasco R, Williams CL. Novel mechanism of the co-regulation of nuclear transport of SmgGDS and Rac1. J Biol Chem. 2003;278(14):12495–506. Epub 20030127. doi: 10.1074/jbc.M211286200 12551911

[54] Mendoza-Catalán MA, Cristóbal-Mondragón GR, Adame-Gómez J, del Valle-Flores HN, Coppe JF, Sierra-López L, et al. Nuclear expression of Rac1 in cervical premalignant lesions and cervical cancer cells. BMC Cancer. 2012;12:116. doi: 10.1186/1471-2407-12-116 22443139 PMC3340301

